# Perspectives in fluid biomarkers in neurodegeneration from the 2019 biomarkers in neurodegenerative diseases course—a joint PhD student course at University College London and University of Gothenburg

**DOI:** 10.1186/s13195-020-00586-6

**Published:** 2020-02-28

**Authors:** Pawel Obrocki, Ayesha Khatun, Deborah Ness, Konstantin Senkevich, Jörg Hanrieder, Federica Capraro, Niklas Mattsson, Ulf Andreasson, Erik Portelius, Nicholas J. Ashton, Kaj Blennow, Michael Schöll, Ross W. Paterson, Jonathan M. Schott, Henrik Zetterberg

**Affiliations:** 1 Department of Medicine, St Mary’s Hospital, Imperial College Healthcare NHS Trust, London, UK; 2 Dementia Research Centre, Department of Neurodegeneration, UCL Institute of Neurology, London, UK; 3 Department of Forensic and Neurodevelopmental Sciences, Institute of Psychiatry, Psychology and Neuroscience, King’s College London, London, UK; 4 First Pavlov State Medical University of St. Petersburg, St. Petersburg, Russia; 5 Petersburg Nuclear Physics Institute named by B.P. Konstantinov of National Research Center, Kurchatov Institute, Gatchina, Russia; 6 Department of Molecular Neuroscience, UCL Institute of Neurology, London, UK; 7 Department of Psychiatry and Neurochemistry, Institute of Neuroscience and Physiology, the Sahlgrenska Academy at the University of Gothenburg, Mölndal, Sweden; 8 The Francis Crick Institute, London, UK; 9 Department of Neuromuscular Diseases, University College London Queen Square Institute of Neurology, London, UK; 10 Clinical Memory Research Unit, Department of Clinical Sciences Malmö, Faculty of Medicine, Lund University, Lund, Sweden; 11 Wallenberg Center for Molecular Medicine, Lund University, Lund, Sweden; 12 Clinical Neurochemistry Laboratory, Institute of Neuroscience and Physiology, the Sahlgrenska Academy at the University of Gothenburg, Mölndal, Sweden; 13 Wallenberg Centre for Molecular and Translational Medicine, Department of Psychiatry and Neurochemistry, the Sahlgrenska Academy at the University of Gothenburg, Gothenburg, Sweden; 14 Institute of Psychiatry, Psychology & Neuroscience, Maurice Wohl Clinical Neuroscience Institute, King’s College London, London, UK; 15 NIHR Biomedical Research Centre for Mental Health & Biomedical Research Unit for Dementia at South London & Maudsley NHS Foundation, London, UK; 16 Clinical Memory Research Unit, Department of Clinical Sciences, Lund University, Malmö, Sweden; 17 UK Dementia Research Institute, University College London, London, UK; 18 Department of Neurodegenerative Disease, University College London Institute of Neurology, London, UK

**Keywords:** Biomarkers, Neurodegeneration, Amyloid, Tau, Neurofilament light, Neurogranin, α-Synuclein, ELISA, Simoa, Mass spectrometry

## Abstract

Until relatively recently, a diagnosis of probable Alzheimer’s disease (AD) and other neurodegenerative disorders was principally based on clinical presentation, with post-mortem examination remaining a gold standard for disease confirmation. This is in sharp contrast to other areas of medicine, where fluid biomarkers, such as troponin levels in myocardial infarction, form an integral part of the diagnostic and treatment criteria. There is a pressing need for such quantifiable and easily accessible tools in neurodegenerative diseases.

In this paper, based on lectures given at the 2019 Biomarkers in Neurodegenerative Diseases Course, we provide an overview of a range of cerebrospinal fluid (CSF) and blood biomarkers in neurodegenerative disorders, including the ‘core’ AD biomarkers amyloid β (Aβ) and tau, as well as other disease-specific and general markers of neuroaxonal injury. We then highlight the main challenges in the field, and how those could be overcome with the aid of new methodological advances, such as assay automation, mass spectrometry and ultrasensitive immunoassays.

As we hopefully move towards an era of disease-modifying treatments, reliable biomarkers will be essential to increase diagnostic accuracy, allow for earlier diagnosis, better participant selection and disease activity and treatment effect monitoring.

## Background

Considerable progress has been made in the field of fluid and imaging biomarker research in neurodegeneration in the last two decades. As a result, the most recent research and clinical guidelines (NIA-AA, IWG-2, NICE) incorporate cerebrospinal fluid (CSF) and positron emission tomography (PET) biomarkers in the diagnostic criteria of Alzheimer’s disease (AD) and mild cognitive impairment (MCI) [1–3]. With more clinical trials of potential disease-modifying treatments shifting the focus towards pre-clinical stages of neurodegenerative disorders, there is an urgent need for more easily accessible, sensitive and specific biomarkers, which could aid earlier diagnosis, patient selection and tracking of disease activity.

The 2019 Biomarkers in Neurodegenerative Diseases Course provided attendants with an opportunity to gain basic and practical knowledge the rapidly developing field. At the 4-day conference aimed at PhD students, an international panel of experts discussed the current state of CSF and blood-derived biomarkers, including emerging technological advances and areas requiring further research. In this paper, based on the course proceedings, we will present a brief overview of the most important fluid biomarkers, focusing on AD, as well as other neurodegenerative disorders. We then outline the current limitations for use and the most recent technological advances in the field.

## Biomarkers in neurodegeneration

### Amyloid and tau

In AD, identification of amyloid β (Aβ) and phosphorylated tau (p-tau) as major components of extracellular plaques and neurofibrillary tangles led to establishment of the core biomarkers for the disease, with a CSF profile characterised by decreased Aβ_42_ levels, and elevated levels of total tau (t-tau) and p-tau (for example at threonine 181) [4]. The reduction in CSF Aβ_42_ levels likely results from selective retention of Aβ_42_ in Aβ plaques, while the increase in t-tau and p-tau levels in CSF reflect increased tau secretion and phosphorylation from neurons affected by AD [4, 5]. A 2016 meta-analysis by Olsson and colleagues comprising over 230 studies helped establish that these biomarkers could help differentiate AD patients from controls, and individuals with MCI with subsequent progression to AD from those with stable MCI [6]. Their high sensitivity and specificity—between 85 and 95% if combined—has led to their incorporation into research guidelines and clinical trials and increasingly into clinical practice in cases when there is a degree of uncertainty about the AD diagnosis [1–3].

#### CSF Aβ

CSF Aβ_42_ is one of the most well-validated biomarkers in neurodegeneration. CSF Aβ_42_ is reduced in MCI patients years before conversion to AD dementia and remains low throughout the disease course [7]. Low CSF levels strongly correlate with cortical amyloid plaque load in the neocortex and hippocampus in post-mortem studies, as well as cortical Aβ deposition measured by PET [8, 9]. More recently, the CSF Aβ_42_/Aβ_40_ peptide ratio has shown to improve prediction of cortical amyloid deposition and differentiation between AD and other dementias in comparison to Aβ_42_ alone, likely by normalising the inter-individual differences in Aβ and release into CSF [10, 11].

In addition to Aβ_42_, numerous studies explored the role of alternatively cleaved Aβ peptides. Aβ_43_, for example, shows comparable diagnostic performance to CSF Aβ_42_ [12]. Yet another Aβ peptide is the shorter Aβ_38_, with research suggesting an association between CSF Aβ_38_ levels and amyloid PET [6, 13].

#### Blood Aβ

Studies implementing novel techniques, such as mass spectrometry and ultrasensitive immunoassays, have shown promise in developing sensitive blood-based Aβ assays [14]. Plasma Aβ_42_ measured using single molecule array (Simoa) technology was shown to be decreased in AD compared with controls and a ratio of plasma Aβ_42_/Aβ_40_ was reduced in amyloid PET positive cases in a manner similar to CSF, but according to most studies, with greater overlap between Aβ-positive and Aβ-negative patients [15, 16]. In contrast to Simoa, two recent papers utilising immunomagnetic reduction (IMR) developed by MagQu have demonstrated increase in plasma Aβ_42_ in AD patients in comparison to controls, which correlated negatively with CSF Aβ_42_ [17, 18]. Significant variability between studies remains an issue, with several potential confounders, including inter-assay differences and potential peripheral Aβ expression contributing to poor concordance and necessitating further validation studies to establish the role of plasma Aβ in AD diagnosis [19].

#### CSF tau

CSF concentrations of t-tau and p-tau are consistently increased in AD [6]. Cognitive decline is more strongly associated with tau pathology than with amyloid pathology, with very high CSF t-tau and p-tau levels associated with worse clinical outcomes [20, 21]. While t-tau and p-tau concentration broadly reflect disease intensity, they correlate poorly with the burden of tau pathology measured by PET or in a post-mortem study [22, 23].

Latest research has focused on the fact that tau proteins can exist in multiple fragments and exhibit different phosphorylation patterns, with hope that some of them might be disease-specific and reflecting the underlying pathophysiological processes. In one study, N-terminal tau fragment truncated at 224 amino acids (N-224) colocalised to neurofibrillary tangles in brain extracts and showed significantly higher levels in CSF from patients with AD in comparison to controls, with higher baseline levels predictive of steeper cognitive decline [24]. More recently, tau N-368 has also been found to be significantly elevated in CSF of AD patients, with a ratio of tau N-368 to total tau exhibiting a strong negative correlation with tau PET [25]. AD pathology also significantly affects phosphorylation patterns, with hyperphosphorylation seen of a number CSF tau sites in comparison to healthy controls. In addition, a distinct phosphorylation site (T153) has been identified in AD CSF, which is absent in non-AD CSF [26].

Interestingly, elevated tau levels, including specific phosphorylated epitopes (P-tau181, P-tau231, and P-tau199) and N-terminal tau fragments truncated at 224, are not seen in many neurodegenerative diseases including primary tauopathies, such as frontotemporal dementia (FTD) or progressive supranuclear palsy (PSP) [24, 27–29]. A recent study by Sato et al. using stable isotope labelling method (SILK) to investigate tau metabolism suggests that the raised t-tau and p-tau levels seen in AD could be due to active production and secretion from neurons in response to Aβ pathology rather than a direct reflection of a neurodegenerative process [30].

#### Blood tau

Plasma t-tau was also found to be increased in AD, though this is not correlated with CSF [31, 32]. Promising results now exist for plasma p-tau, measured using a sensitive immunoassay with electrochemiluminescence detection and showing strong association with tau PET, as well as high concordance with CSF p-tau in a recent study by Palmqvist et al. [33, 34]. Several large replication studies, showing robust correlations with CSF p-tau and amyloid PET results, were presented during Alzheimer’s Association International Conference 2019 (AAIC) but have not yet been published.

In conclusion, while raised CSF tau levels are a well-validated feature of AD, studies examining the biology of tau, including its processing, secretion and aggregation are needed to fully understand its role as an AD biomarker. There is also a need for further research on tau pathology biomarkers in other tauopathies, such as PSP.

### Neurofilament light

Neurofilament light (NfL) is a type of intermediate filament seen in the cytoplasm of axons, where it plays an important role in axonal homeostasis and synaptic transmission [35]. NfL concentrations dynamically increase in response to concussion, as demonstrated in amateur boxers and ice hockey players [36, 37]. NfL has also been used as a biomarker of disease intensity, since it correlates with neuroaxonal damage in a wide range of neurological disorders [38]. Importantly, CSF and serum NfL concentrations are highly correlated, hence they will be discussed together [39, 40].

#### CSF and blood NfL

Serum NfL concentration is increased in familial AD a decade prior to symptom onset and correlates with degree of whole-brain atrophy seen on magnetic resonance imaging (MRI) and cognition [41–43]. In sporadic AD, high plasma NfL levels distinguish between AD, MCI and healthy controls, with higher values among MCI subjects associated with more rapid brain atrophy [44]. Plasma NfL also associates with the degree neurofilament staining and Braak staging at post-mortem [45]. Longitudinal increase in plasma NfL positively correlates with longitudinal changes in other measures of neurodegeneration, including brain atrophy and cognition [46].

NfL is an useful biomarker in other forms of neurodegeneration. CSF NfL level has been shown to differ between AD and other forms of dementia—for example, FTD patients exhibit significantly higher values of CSF NfL in comparison to AD patients, as reported in recent post-mortem study [47]. Serum NfL can also discriminate between idiopathic Parkinson’s disease (PD) and atypical parkinsonism that is clinically indistinguishable at the stage of testing [48, 49]. In Huntington’s disease (HD), plasma NfL levels are closely associated with MRI brain volume and clinical severity and may be a useful outcome measure in tracking clinical response to disease-modifying therapies [50]. High levels of NfL are also seen in other neurodegenerative disorders, such as amyotrophic lateral sclerosis (ALS), HIV-associated dementia (HAD) and Creutzfeldt-Jakob disease (CJD) [51]. In addition to very high NfL levels seen in CJD, the rapidly progressive disease exhibits unique, multi-fold increase in concentration of multiple other CSF biomarkers, including total tau, alpha-synuclein and neurogranin [52–54].

The role of NfL as a biomarker extends beyond the scope of neurodegeneration, with multiple sclerosis (MS), a common neuroinflammatory central nervous system (CNS) disorder being a prominent example. The levels of NfL are significantly increased in patients with MS versus healthy controls, and positively correlate with the burden of disease activity seen on MRI [55, 56]. Conversely, reduction of NfL concentration is seen in MS patients who commence disease-modifying treatment, or switch from first-line to a more high-potency treatment [57].

Taken together, the data suggests that CSF, serum and plasma NfL is a sensitive, but non-specific marker of disease activity in the CNS and peripheral nervous system (PNS), with additional benefit of being able to measure disease activity and severity, as demonstrated in MS and HD, as well as treatment response, as shown in MS or spinal muscular atrophy (SMA) [58, 59].

### Neurogranin

It has been widely shown that synaptic dysfunction occurs at early stages of AD, predating the onset of overt neuronal loss [60]. Neurogranin (Ng), a calmodulin-binding postsynaptic protein, is highly expressed in brain regions important in memory processing, such as amygdala and hippocampus, where it plays a crucial role in long-term potentiation [61].

#### CSF neurogranin

Since its discovery in the CSF, multiple studies have shown that Ng is increased in AD and MCI patients compared to controls and that higher levels are predictive of a steeper degree of cognitive decline, a reduction in cortical glucose metabolism and hippocampal volume loss [62].

The elevation of CSF Ng seems to be specific for AD and is not seen in other neurodegenerative disorders beside CJD [52, 63, 64]. A recent study examining post-mortem parietal and temporal cortex tissues found that the ratio of peptide-to-total full-length Ng was higher in patients with AD compared to controls, suggesting increased processing of Ng into peptides [65]. Thus, the mechanisms underlying CSF Ng increase in AD could be similar to those of increased CSF tau processing and release in the disease [30].

#### Blood neurogranin

Few studies have investigated plasma Ng levels and failed to show a significant difference between AD patients and healthy controls; however, pilot studies showed that the concentration of Ng from neuron-derived exosomes is lower in AD in comparison to controls and was associated with progression from MCI to AD [66, 67].

Altogether, the current evidence shows that Ng is a promising biomarker reflecting early synaptic dysfunction in AD, which can have a predictive value in healthy controls as well as MCI patients, in a surprisingly AD-specific manner.

### α-Synuclein

α-Synuclein is a short cytoplasmic protein implicated in synaptic transmission and intracellular trafficking [68]. Misfolding and aggregation of α-synuclein into oligomers and fibrils, with prion-like seeding throughout the CNS is believed to be central to the pathogenesis of a range of neurodegenerative disorders, including PD, LBD and multiple system atrophy (MSA) [68, 69]. It has been shown that α-synuclein is detectable in a range of biofluids, such as CSF, serum, saliva or tears [70].

#### CSF α-synuclein

Total α-synuclein is the most well studied in CSF, with a meta-analysis showing that the concentrations in patients with synucleinopathies are lower than those of healthy controls [71]. However, results are neither sensitive nor specific enough to allow for use of the biomarker for diagnostic purposes, with evidence of significant inter-subject and inter-laboratory variation, complicated by the fact that blood contamination of the CSF could significantly raise total α-synuclein concentration [72]. In addition, one study suggested that PD patients with an aggressive clinical course tend to have higher baseline α-synuclein concentration, complicating the interpretation [73]. In contrast to PD, CSF α-synuclein levels were found to be raised in AD, with extremely high levels reported in CJD [74].

More recently, studies utilising prion-like properties of α-synuclein by measuring a degree of protein aggregation using real-time quaking-induced conversion assay (RT-QuiC) accurately distinguished between neuropathologically confirmed cases of PD or LBD and controls, with 92–95% sensitivity and 100% specificity [75, 76]. Interestingly, one study demonstrated significant α-synuclein aggregation in two control subjects who then went on to develop PD years after the sample was obtained [77].

In addition to total CSF α-synuclein, levels of the CSF oligomeric and phosphorylated α-synuclein have both been reported to be elevated in PD compared to controls, which requires further validation [78].

#### Blood α-synuclein

The protein is widely expressed in multiple fluids outside of the CNS, with red blood cells being a major source of α-synuclein in the blood and a source of potential contamination [70, 79]. Trials measuring α-synuclein in whole blood, plasma and serum of PD patients yielded conflicting results, limiting its utility as a diagnostic biomarker [78]. However, similarly to CSF, studies measuring oligomeric or phosphorylated forms of the protein in the serum and in red blood cells have shown to be consistently elevated in PD patients in comparison to controls [78, 80].

Currently, α-synuclein remains one of the most complex biomarkers to interpret due to several potential confounding factors. Further research into aggregation assays, as well as oligomeric and Lewy body-enriched forms of the protein, is needed to establish it as a clinically useful biomarker.

### Other biomarker candidates

TAR DNA-binding protein 43 (TDP-43) cytoplasmic accumulation is characteristic feature of ALS and FTD [81]. TDP-43 pathology is also present in 20–50% of AD cases, but the protein is difficult to detect in body fluids and CSF TDP-43 seems to be primarily blood-derived [82]. In one study, CSF TDP-43 was raised in ALS and FTD versus healthy controls, but considerable overlap between the groups was seen [83]. Another paper showed raised plasma TDP-43 levels in a proportion of FTD and AD patients (46% and 22%, respectively) in comparison to controls [84]. Currently, there are no fluid-based assays specific for pathological forms of the protein.

Inflammation contributes to AD pathogenesis and proteins involved in the inflammatory response, such as triggering receptor expressed on myeloid cells 2 (TREM2) and YKL-40 (also known as chitinase-3-like protein 1), could be used as potential AD biomarkers. TREM2 is expressed in microglia, and its soluble form is upregulated in the CSF of MCI and AD patients [85, 86]. YKL-40 is expressed in astrocytes, with CSF showing increased concentration in AD and predictive of progression of MCI to AD [87]. Higher levels have also been shown to correlate with burden of tau pathology [88].

β-Site APP-cleaving enzyme 1 (BACE1) is an endoprotease closely involved in amyloid precursor protein (APP) processing. CSF BACE1 levels have been demonstrated to be higher in MCI and AD in comparison to healthy controls, especially in the presence of APOE ε4 allele [89, 90]. In another study, plasma BACE1 levels were able to indicate future MCI to AD progressors [91].

In addition to Ng, other synaptic proteins, such as synaptotagmin-1 (SYT-1), synaptosomal-associated protein-25 (SNAP-25) and growth-associated protein-43 (GAP-43), have been detected in CSF of AD patients and are a promising group of biomarkers, highlighting the importance of synaptic dysregulation in the disease [92–94].

## Current limitations and future perspectives

### CSF sampling

In the field of neurodegeneration, most progress has been made with CSF biomarkers. Lumbar puncture is considered to be a safe and is generally well tolerated procedure, but its use can be limited by certain contraindications (e.g. taking anticoagulants), patient non-compliance or lack of resources [95]. More accessible biofluids, such as blood or urine, would undoubtedly improve access to sample material and facilitate access to repeated longitudinal samples that could be valuable for tracking disease progression. However, concentration of CNS biomarkers outside of CSF is often extremely low, making it difficult to detect using standard assays. Other important factors complicating the analysis include peripheral expression of the protein of interest, endogenous antibodies interfering with assay results and presence of proteases which shorten the lifespan of the protein in peripheral tissues [96].

### Sources of variation

The gold standard method for measuring CSF Aβ_42_ and tau is with enzyme-linked immunosorbent assays (ELISA). The methods of handling and storing CSF samples can differ between centres, and certain factors can be of critical importance. For example, storage tube material, aliquot volume and the number of consecutive tube transfers the sample is subjected to can significantly impact the measured biomarker concentration [97]. Variation in CSF measures is also observed within assays and between centres. Interlaboratory coefficients of variation (CVs) are observed at 20–30% whereas intra-laboratory studies report CVs of < 10% [98–100]. Initiatives taken to improve analytical standardisation between centres were discussed, including the introduction of a certified reference materials for assay standardisation, an external quality control programme and the use of fully automated ELISA platforms, which has reduced intra- and inter-laboratory variation considerably (from 10 to 20% to 1–5%) [98, 101–104].

### Ultrasensitive immunoassays

As a result of a selectivity of the blood-brain barrier, as well as high blood to CSF volume ratio, the concentration of CNS-derived proteins in blood is much less than in the CSF. The sensitivity of ELISA is therefore not high enough to identify and reliably quantify the concentration of CNS biomarkers in the plasma or serum. However, a number of ultrasensitive immunoassays with superior analytical sensitivity now exist, including Simoa (Quanterix), single molecule counting (SMC by Merck), proximity extension assay (OLINK) and immunomagnetic reduction (IMR by MagQu) [105].

With increased availability of ultrasensitive immunoassays, blood biomarkers hold promise for the future as less-invasive, cost-effective screening tests for neurodegenerative disorders [16].

### Mass spectrometry

Another approach to study biomarkers is mass spectrometry (MSp)-based tests, which allow for quantification and characterisation of peptides in a wide range of biofluids, including CSF and serum. MSp combines good sensitivity and specificity, high multiplexing capacity and the ability to detect proteins which have been post-translationally modified or truncated [106].

Two MSp-based reference methods for CSF Aβ_42_ have been certified by the Joint Committee for Traceability in Laboratory Medicine [107, 108].

Recent research utilising mass spectrometry (MSp) in the field of AD blood biomarker discovery have also shown promising results. A study by Kaneko et al. reported an almost 90% diagnostic accuracy in classifying amyloid PET positive and non-positive individuals using the ratio of a specific APP fragment (APP669-711) in plasma to plasma Aβ_42_ level [109]. More recently, studies using a more sensitive IP-MSp method detected a decreased Aβ_42_/Aβ_40_ ratio in plasma and reported a diagnostic accuracy of almost 90% of plasma Aβ_42_ /Aβ_40_ ratio in predicting Aβ PET positivity in AD, MCI and cognitively normal states [110]. Similar results have been presented by other groups [111, 112]*.*

This approach represents a potentially cost-effective and accessible way of measuring Aβ burden in an individual; however, further validation and longitudinal studies, as well as standardisation across institution, are needed before potential clinical application of the promising MSp approach.

### Proteomics, metabolomics and lipidomics

With the advent of novel proteomic techniques, proteomics-based approaches have become an important tool in biomarker discovery that can complement genomic analysis and provide important clues to the pathophysiology of many neurodegenerative disorders. An example of a new proteomics tool used in the field includes proximity extension assay developed by Olink Proteomics AB, which offers high sensitivity and multiplexing ability [113]. A recent large study, utilising Olink measuring 270 CSF and plasma proteins in AD patients, identified significant differences in the concentrations of 10 CSF and 6 plasma proteins that take part in a variety of biological processes, including inflammation and apoptosis [114]. In addition, plasma biomarkers were able to distinguish between AD, prodromal AD and healthy controls with high accuracy. In another study focusing on atypical parkinsonian syndromes (APS), 11 novel CSF proteins involved were identified that significantly differed between APS patients and healthy controls, with 4 protein levels also distinguishing between APS and PD patients [115]. The identified proteins are involved in a variety of cellular processes, including cell proliferation and immune cell migration.

Metabolomics and lipidomics have emerged as promising approaches for the comprehensive study of complex biological samples and for biomarker discovery [116, 117]. There have been significant efforts to characterise metabolites and lipids in neurodegeneration, with hope that the observed lipid and metabolite profiles reflect metabolic changes and lipid-mediated mechanisms associated with disease pathology. These may serve as characteristic fingerprints of disease state and could potentially reveal therapeutic targets [118].

Over the past decade, targeted and non-targeted approaches for metabolomics/lipidomics have been significantly improved, largely due to improvements of MSp instrumentation [116, 117]. Several metabolomic studies have been reported in the context of AD biomarker discovery, most prominently using a commercial assay for targeted metabolite and lipid quantification in blood [119–125]. However, follow-up studies failed to replicate the findings, which fuelled the Alzheimer Disease Metabolomics Consortium (ADMC) initiative, where a network approach is used to establish a common metabolomic database of AD [126, 127].

A recent study reported that levels of primary fatty amides in plasma associated with CSF Aβ and hippocampal volume on MRI [128]. Another metabolomic study on serum bile acid (BA) profiles in AD showed that serum-based BA metabolites are associated with CSF Aβ and p-tau [129]. These examples highlight that well designed both targeted and untargeted metabolomic and lipidomic studies can reveal new biomarkers for AD pathology and improve our mechanistic understanding of AD pathophysiology.

## Conclusions

The Biomarkers in Neurodegenerative Diseases Course provided delegates with an overview of the fluid biomarker field. There are now core biomarkers of neurodegenerative pathology (amyloid, tau and α-synuclein), a biomarker of disease intensity (NfL), synaptic function (neurogranin) and a range of novel analytical platforms such as Simoa and MSp. Future challenges include refining pre-analytical and analytical standardisation, measuring other aspects of neurodegenerative pathophysiology and developing less-invasive fluid biomarkers that can also be used for screening and tracking purposes.

## Abbreviations

Aβ: Amyloid β
AAIC: Alzheimer’s Association International Conference
AD: Alzheimer’s disease
ADMC: Alzheimer Disease Metabolomics Consortium
ALS: Amyotrophic lateral sclerosis
APP: Amyloid precursor protein
BA: Bile acid
BACE1: β-site APP-cleaving enzyme 1
CJD: Creutzfeldt-Jakob disease
CNS: Central nervous system
CSF: Cerebrospinal fluid
CV: Coefficient of variation
ELISA: Enzyme-linked immunosorbent assay
GAP-43: Growth-associated protein-43
HAD: HIV-associated dementia
HD: Huntington’s disease
IWG-2: International Working Group 2
MCI: Mild cognitive impairment
MRI: Magnetic resonance imaging
MS: Multiple sclerosis
MSA: Multisystem atrophy
MSp: Mass spectrometry
NfL: Neurofilament light
Ng: Neurogranin
NIA-AA: National Institute of Aging and Alzheimer’s Association
NICE: The National Institute for Health and Care Excellence
P-tau: Phosphorylated tau
PD: Parkinson’s disease
PET: Positron emission tomography
PNS: Peripheral nervous system
PSP: Progressive supranuclear palsy
RT-QuiC: Real-time quaking-induced conversion assay
Simoa: Single molecule array
SMA: Spinal muscular atrophy
SNAP-25: Synaptosomal-associated protein-25
SYT-1: Synaptotagmin-1
TDP-43: TAR DNA-binding protein 43
TREM2: Triggering receptor expressed on myeloid cells 2
T-tau: Total tau
YKL-40: Chitinase-3-like protein 1
